# Protein flexibility in the light of structural alphabets

**DOI:** 10.3389/fmolb.2015.00020

**Published:** 2015-05-27

**Authors:** Pierrick Craveur, Agnel P. Joseph, Jeremy Esque, Tarun J. Narwani, Floriane Noël, Nicolas Shinada, Matthieu Goguet, Sylvain Leonard, Pierre Poulain, Olivier Bertrand, Guilhem Faure, Joseph Rebehmed, Amine Ghozlane, Lakshmipuram S. Swapna, Ramachandra M. Bhaskara, Jonathan Barnoud, Stéphane Téletchéa, Vincent Jallu, Jiri Cerny, Bohdan Schneider, Catherine Etchebest, Narayanaswamy Srinivasan, Jean-Christophe Gelly, Alexandre G. de Brevern

**Affiliations:** ^1^Institut National de la Santé et de la Recherche Médicale U 1134Paris, France; ^2^UMR_S 1134, DSIMB, Université Paris Diderot, Sorbonne Paris CiteParis, France; ^3^Institut National de la Transfusion Sanguine, DSIMBParis, France; ^4^UMR_S 1134, DSIMB, Laboratory of Excellence GR-ExParis, France; ^5^Rutherford Appleton Laboratory, Science and Technology Facilities CouncilDidcot, UK; ^6^Institut National de la Santé et de la Recherche Médicale U964,7 UMR Centre National de la Recherche Scientifique 7104, IGBMC, Université de StrasbourgIllkirch, France; ^7^Ets PoulainPointe-Noire, Congo; ^8^National Library of Medicine, National Center for Biotechnology Information, National Institutes of HealthBethesda, MD, USA; ^9^Centre National de la Recherche Scientifique UMR7590, Sorbonne Universités, Université Pierre et Marie Curie – MNHN – IRD – IUCParis, France; ^10^Metagenopolis, INRAJouy-en-Josas, France; ^11^Molecular Biophysics Unit, Indian Institute of Science, BangaloreBangalore, India; ^12^Hospital for Sick Children, and Departments of Biochemistry and Molecular Genetics, University of TorontoToronto, ON, Canada; ^13^Department of Theoretical Biophysics, Max Planck Institute of BiophysicsFrankfurt, Germany; ^14^Laboratoire de Physique, École Normale Supérieure de Lyon, Université de Lyon, Centre National de la Recherche Scientifique UMR 5672Lyon, France; ^15^Faculté des Sciences et Techniques, Université de Nantes, Unité Fonctionnalité et Ingénierie des Protéines, Centre National de la Recherche Scientifique UMR 6286, Université NantesNantes, France; ^16^Platelet Unit, Institut National de la Transfusion SanguineParis, France; ^17^Institute of Biotechnology, The Czech Academy of SciencesPrague, Czech Republic

**Keywords:** protein structures, disorder, secondary structure, structural alphabet, protein folding, allostery, protein complexes, protein—DNA interactions

## Abstract

Protein structures are valuable tools to understand protein function. Nonetheless, proteins are often considered as rigid macromolecules while their structures exhibit specific flexibility, which is essential to complete their functions. Analyses of protein structures and dynamics are often performed with a simplified three-state description, i.e., the classical secondary structures. More precise and complete description of protein backbone conformation can be obtained using libraries of small protein fragments that are able to approximate every part of protein structures. These libraries, called structural alphabets (SAs), have been widely used in structure analysis field, from definition of ligand binding sites to superimposition of protein structures. SAs are also well suited to analyze the dynamics of protein structures. Here, we review innovative approaches that investigate protein flexibility based on SAs description. Coupled to various sources of experimental data (e.g., B-factor) and computational methodology (e.g., Molecular Dynamic simulation), SAs turn out to be powerful tools to analyze protein dynamics, e.g., to examine allosteric mechanisms in large set of structures in complexes, to identify order/disorder transition. SAs were also shown to be quite efficient to predict protein flexibility from amino-acid sequence. Finally, in this review, we exemplify the interest of SAs for studying flexibility with different cases of proteins implicated in pathologies and diseases.

## Introduction

Analysis of protein structures is crucial to understand protein dynamics and functions. X-ray crystallography, the gold-standard method for solving 3D structures at atomic resolution, is impeded by protein dynamics. Hence, tricks are frequently used to restrict motions. It is why proteins have been often considered as static macromolecules, composed of *rigid* repetitive secondary structures and *less rigid* random coils. However, more and more emerging evidences show that protein structures are more complex with their internal dynamics being a key determinant of their function. Analyses of protein structures are often performed with a simplified three-state description known as α-helix, β-strand and coil which constitutes the classical secondary structures (Corey and Pauling, 1953; Kabsch and Sander, 1983). A more precise and complete description of protein backbone conformation exists based on the definition of libraries of small protein fragments, namely the structural alphabets (SAs) (Unger et al., 1989; Fetrow et al., 1997; Camproux et al., 1999; Offmann et al., 2007; Tyagi et al., 2007; Joseph et al., 2010a,b). SAs are designed to approximate every part of the local protein structures providing conformational detail. They have performed remarkably well spanning various problems in structural bioinformatics, from the characterization of ligand binding sites to the superimposition of protein structures (Joseph et al., 2010b). Furthermore, SAs are also very well suited to analyze the internal dynamics of protein structures. SAs have been used at three different levels to comprehend protein flexibility: (i) for studying specific fundamental biological and biomedical problems, (ii) to analyze changes associated with protein complexation and allostery, and (iii) to predict protein flexibility.

Here, we present state-of-the-art of developments in the study of protein flexibility using SAs based approximation. The backbone conformational variations can be described as changes in the pattern of SAs, which acts as fingerprints of the dynamics involved. These innovative approaches are useful, customizable, and deal with specific proteins involved in pathologies and diseases. They are also powerful to evaluate generalized principles from large biological complex structures. Thus, SAs provide new vision for detailed analysis and prediction flexibility of proteins.

## The different views of protein structures

**Figure 1:**
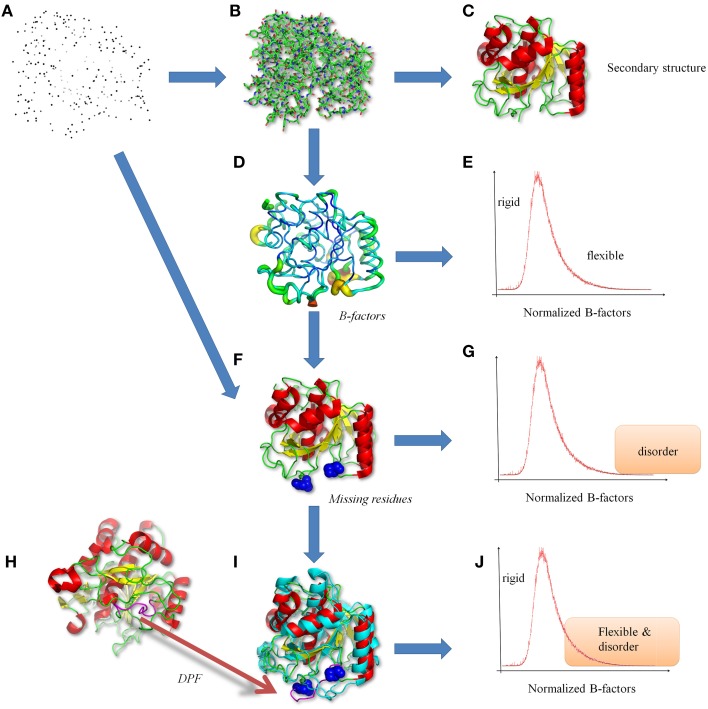
**Classical views of protein structures**. **(A)** The protein structure is a file in PDB format (Bernstein et al., 1977; Berman et al., 2000), containing the 3D atomic coordinates. **(B)** The atoms are bound to build the protein backbone and side-chain residues. **(C)** From this information, secondary structures are performed (Kabsch and Sander, 1983). **(D)** From crystallographic data, B-factors are analyzed underlining **(E)** rigid to flexible residues. **(F)** More precise analyses shows missing residues revealing. **(G)** disorder regions (Uversky et al., 2000; Dunker et al., 2001). **(H)** Interestingly same or similar proteins can be found in the PDB (Berman et al., 2000), and **(I)** in numerous times with the missing regions resolved, leading to **(J)** a more complex definition and an ambiguity between flexibility and disorder. Protein visualization was created by the program PyMOL (http://www.pymol.org, Delano, 2013). The proteins used are two proteases (PDB codes 1dbi chain A, and 1wmd chain A, for this last only residues 1–306 are shown for more clarity).

The primary sequence of the protein—the succession of amino acids—is assumed to encompass all the information necessary for its function. The protein structures resolved from X-ray crystallography or Nuclear Magnetic Resonance (NMR) (see Figures 1A,B) can be obtained in the Protein DataBank format (PDB, Bernstein et al., 1977; Berman et al., 2000). From the very beginning, theoreticians or experimentalists have described local protein structures by using three states (see Figure 1C, Corey and Pauling, 1953; Kabsch and Sander, 1983; Eisenberg, 2003). Two of them are repetitive structures stabilized by hydrogen bond patterns, namely the α-helices and the β-sheets (composed of β-strands). These structures are connected with more variable structures, i.e., random coil or loops. Later studies have identified spotted small repetitive and regular structures such as the β-hairpins or different kinds of turns in several protein structures (Richardson, 1981). These simplified descriptions were nicely represented with 3D visualization software (e.g., arrows for β-sheets, springs for α-helix) and accompanying the emergence of macromolecular crystallography. However these simplistic representations also contributed to the static and rigid views of these structures (Chavent et al., 2011).

In fact, growing evidence shows that proteins are highly dynamic macromolecules and that this dynamics is crucial in many biological processes. Thus, recent studies have demonstrated that conformational transitions in folded states of many proteins are essential to accomplish their functions, e.g., enzyme catalysis, activity regulation (Goh et al., 2004; Grunberg et al., 2004; Lensink and Mendez, 2008). Flexibility also allows interactions with different partners, with ligands by induced-fit interaction, with other proteins, or nucleic acids to form complex structures. NMR based methods and computational experiments such as Molecular Dynamic (MD) simulations, have largely contributed to gain valuable insights into the observation, understanding, and analyses of flexibility (Hirst et al., 2014). Flexibility can be versatile and covers a large range of timescales and amplitudes of structural modifications. It encompasses different kinds of conformational changes corresponding to (i) mobility of rigid part of the protein, e.g., domain motions (ii) deformability of the protein backbone, e.g., crankshaft motions or (iii) both. These different transitions are shown by analyzing and comparing protein structures (see Figure 1D). At a local level, the flexibility can be identified by the information contained in diffraction images of X-ray crystallography experiments and quantified along the refinement process through the Debye-Waller factors (expressed as surface units) also known as “B-factors” or temperature (displacement) factors. These so-called B-factors reflect atom mobility due to thermal vibration and measure the static disorder. They allow quantifying different levels of flexibility in proteins (see Figure 1E, Marsh, 2013). This criterion is also used by majority of flexibility prediction methods (from the sequence) (Schlessinger and Rost, 2005).

In this context, missing coordinates of whole residues in X-ray protein structures (usually labeled as missing residues, see Figure 1F) and several dedicated biochemical analyses have suggested these protein segments should be considered as disordered regions (see Figure 1G, Uversky et al., 2000; Dunker et al., 2001). From few years, beside the paradigm of a well-defined 3D folded state, new visions of protein structure and dynamics have emerged, namely the Intrinsically Disordered Proteins (IDP) or disordered regions. IDP may exhibit large structural rearrangements like the formation (then the loss) of secondary structures depending on the environment or the interacting partners. The impressive amount of research in this field is motivated by the implication of IDP in multiple crucial biological functions (Dunker et al., 2000; Dunker and Obradovic, 2001), for e.g., 14-3-3 proteins (Uhart and Bustos, 2014) or the Innate Antiviral Immunity (Xue and Uversky, 2014). Nevertheless, the regions with missing residues can be found resolved in other PDB structures of the same (or highly homologous) protein (see Figure 1I) (see Figure 1H, Berman et al., 2000). These ambiguous regions, termed Dual Personality Fragments (DPFs, see Dunker, 2007; Zhang et al., 2007), complicate the distinction and *per se* the definition of disorder versus flexibility (see Figure 1J). In Figure 1, we show a protease (PDB code 1dbi chain A), the corresponding DPF found (with a good resolution) in another protease (PDB code 1wmd chain A). Correlation between B-factors (representing flexibility) and disorder predictor outputs has been explored and shows a good agreement (Jin and Dunbrack, 2005; Schlessinger et al., 2009).

In the light of the above observations, the classic representation of protein structure as a succession of repetitive ordered secondary structures and random coil does not allow understanding of the complexity associated with structural flexibility. Actually, the coarseness of the secondary structure assignment may prevent from identifying conformational changes. Therefore distinction between flexible loops and rigid loops, for example, cannot be made on the sole basis of a three-state secondary structure assignment. A more precise and local description of protein structure is needed. In this regard, Structural Alphabet (SAs), allow to investigate primarily the complexity of the protein conformations, and consequently of their associated dynamics.

A SA is a library of *N* structural prototypes (the letters). Each prototype is representative of a backbone local structure of *l*-residues length. The combination of those structural prototypes is assumed to approximate any given protein structure. Many different libraries have been developed, (e.g., Unger et al., 1989; Fetrow et al., 1997; Camproux et al., 1999; Tung et al., 2007). Depending on the targeted accuracy, the length *l* and the number *N* can vary significantly. The length *l* typically ranges between 4 and 9 while can vary, the most frequent value being close to 20 (see Offmann et al., 2007; Joseph et al., 2010a,b for more details). The various structural alphabets also differ by the description parameters of the protein backbone. The description can be based on Cα coordinates, Cα-Cα distances, α or dihedral angles. The classification and learning methods that were used, are also various, e.g., hierarchical clustering, empirical function, Kohonen Maps, neural network or Hidden Markov Model Besides their interest to provide a finer description, They SA have been also designed for prediction purpose, which requires to decipher the sequence—structure relationship.

As example, in their respective work, Park and Levitt (1995) and Kolodny et al. (2002) aimed at finding representations based on smallest libraries of protein fragments to accurately construct protein structures. Fragments of four to seven residues long were considered in a library of 25–300 fragments. Micheletti et al. (2000) did similar studies and constructed a library that encompassed from 28 to 2561 recurrent local structures.

**Figure 2:**
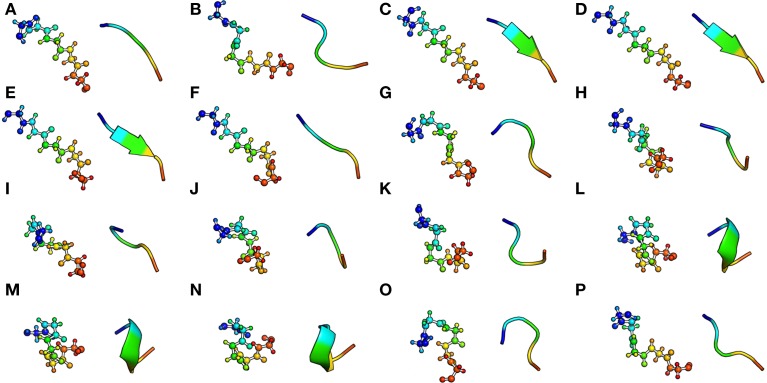
**The Protein Blocks structural alphabet**. The conformation of each 16 pentapeptides is presented with ball and stick (left) and cartoon representation (right). The N-ter and C-ter extremity are respectively colored in blue and red. Visualization was created by the program PyMOL (http://www.pymol.org, Delano, 2013).

To date, one of the most developed and comprehensive SA is the Protein Blocks approach (PBs, de Brevern et al., 2000). This SA is composed by 16 local structure prototypes of 5 residues fragments (see Figure 2). It was shown to efficiently approximate every part of the protein structure. The PBs *m* and *d* can be roughly described as prototypes for the central region of α-helix and β-strand, respectively. PBs *a*-*c* primarily represent the N-cap of β-strand while *e* and *f* correspond to C-caps; PBs *g* -*j* are specific to coils, PBs *k* and *l* correspond to N cap of α-helix while PBs *n*-*p* to C-caps. PBs have been used to address various problems, including protein superimposition (Gelly et al., 2011; Joseph et al., 2012), general analyses of flexibility (Dudev and Lim, 2007; Wu et al., 2010) or and prediction of structure and flexibility (Zimmermann and Hansmann, 2008; Rangwala et al., 2009; Suresh et al., 2013; Joseph and de Brevern, 2014).

**Figure 3:**
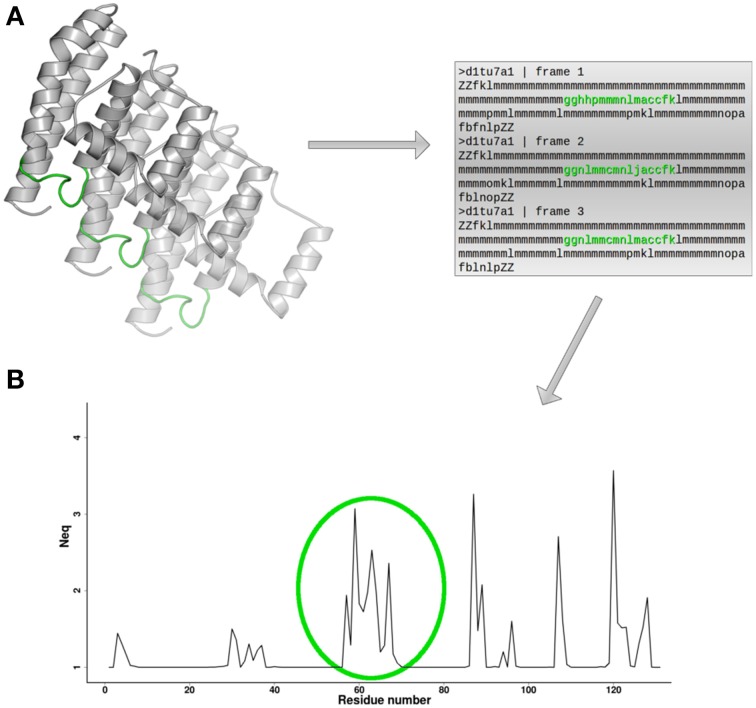
***N*****_eq_ and local flexibility. (A)** For each conformation extracted from MD simulation, a PB sequence is assigned. **(B)** *N*_eq_ profile provides direct identification of protein fragments in which local conformational change is observed. Here, in green, is indicated a flexible loop. The protein 3D structure representation is generated using PyMOL software (http://www.pymol.org, Delano, 2013).

The assignment algorithm (see Figure 3A, de Brevern et al., 2000) runs through the 3D structure of the target protein, from the N to the C-ter of the sequence. The algorithm is iterative and uses 5 residues long overlapping windows over the entire sequence to assign a PB to every position. For each “*n*^th^” position of the structure, 8 dihedrals ψ (*n* − 2), φ (*n* − 1), ψ (*n* − 1), φ (*n*), ψ (*n*), φ (*n* + 1), ψ (*n* + 1), φ (*n* + 2) are compared to each of the 16 PBs. The comparison is made by a least squares approach to match the RMSDA criteria (*Root mean square Deviation on Angular Values*) (Schuchhardt et al., 1996):

(1) $$\begin{array}{l} RMSDA\;(V_{1},V_{2})=\sqrt{\frac{1}{2(M-1)}\sum_{i=1}^{i=M-1}{\left[\psi_{i}(V_{1})-\psi_{i}(V_{2})\right]}^{2}}\\ \;+{\left[\varphi_{i+1}(V_{1})-\varphi_{i+1}(V_{2})\right]}^{2}\\ \text{ RMSDA formula} \end{array}$$

where *V*_1_ is the vector of 8 dihedral angles extracted from the 5 residues long window, and *V*_2_ is the 8 vector of dihedral corresponding to the individual PB type. The PB with the lowest RMSDA, is assigned to the corresponding position for that window. This PB captures the overall local conformation and approximates the transition along the main-chain smoothly.

PB assignments can be done using the Python PBxplore tool (https://github.com/pierrepo/PBxplore, in preparation). The result is a translation of a 3D structure into a 1D sequence of PBs.

Interestingly, the subtle differences between protein conformations can be captured by the assignment of the PB sequences. By analyzing the variation of PBs assigned at a given position for multiple conformers, the local conformational properties and corresponding changes can be easily identified. Moreover, a quantification of the flexibility at a given position *n* can be obtained by calculating, the average number of PBs across a set of conformers in this position or the “equivalent number” of PBs (*N*_eq_). *N*_eq_ is based on a statistical metric similar to Shannon entropy (de Brevern et al., 2000) and is calculated as follows:

(2) $$\begin{array}{l} N_{\text{eq}}=\exp(-\sum_{x\;=\;1}^{16}f_{x}\;ln\;(f_{x}))\\ N_{\text{eq}}\text{formula} \end{array}$$

where *f*_x_ is the frequency of PB *x* (*x* takes values from *a* to *p*). A *N*_eq_ value of 1 indicates that only one type of PB is observed, while a value of 16 is equivalent to a random distribution. For example *N*_eq_ value equal to 6, could mean that 6 different PBs are observed in equal proportions (1/6), or that more than 6 PBs are observed in different proportions. By plotting the computed value for each residue position (see Figure 3B), it is possible to easily localize which protein regions present local conformation change, or in other words, which regions represent local flexibility.

This PB derived-entropy index is an interesting feature of PBs, which can be used to analyze PB prediction (de Brevern et al., 2000) or an ensemble of structures, corresponding to the same protein solved in different experiments, or to several structures extracted from MD simulation (Jallu et al., 2012). Note that PBxplore can be used to calculate *N*_eq_, and to visualize in various ways the PB variation for each position from a collection of models or through a MD trajectory (de Brevern et al., 2005).

*Other interesting SAs used in the flexibility context.* We have proposed an extension of our SA through a novel library consisting of 120 overlapping structural classes of 11-residues fragments, firstly defined as PBs series (Benros et al., 2006). This library was constructed with an original unsupervised structural clustering method called the Hybrid Protein Model (de Brevern and Hazout, 2003). For each class, a mean representative fragment, or “local structure prototype” (LSP), correctly approximate the local structures with an average Cα RMSD of 1.61 Å. LSPs capture both the continuity between the identified recurrent local structures and long-range interactions. From this description, two methodologies were developed to predict flexibility. The first one was based on simple logistic functions and supervised with a system of experts (Benros et al., 2006). The second one was a combination of Support Vector Machines (SVMs) and evolutionary information (Bornot et al., 2009).

Pandini and co-workers developed their own SA; it is derived from the notion of attractors in conformational space, a more complex approach than PBs (Pandini et al., 2010). Pandini and co-workers developed their own SA; it is derived from the notion of attractors in conformational space, a more complex approach than PBs (Pandini et al., 2010). They focused on four-residue long fragments, the conformation of each being defined by internal angles between Cα atoms, i.e., *two* pseudo-bond angles and one pseudo torsion angle. All protein fragments were mapped as points in a three-dimensional space of these internal angles. The optimal number of clusters, i.e., structural prototypes, was assessed by the quality of the reconstructed protein structures and by information content. They ended with an alphabet of 25 letters, called M32K25. The alphabet starts from extended structures (e.g., A letter) and ends with turns (e.g., Y letter), passing through loops (e.g., P letter) and helical structures (e.g., U letter). The authors compared their approach with other SAs of four-residue fragments and showed the superiority of their method (Camproux et al., 2004; Tung et al., 2007). An interesting point was the analysis of the correlation between local flexibility and variability in the assignment. Thereafter, they have developed GSATools, (http://mathbio.nimr.mrc.ac.uk/wiki/GSATools, Pandini et al., 2013), composed of a set of programs, that encode ensembles of protein conformations into alignments of structural strings using their Structural Alphabet. This software package is particularly well suited for the investigation of the conformational dynamics of local structures, the analysis of functional correlations between local and global motions, and the mechanisms of allosteric communication. It performs a wide range of statistical analyses using a various set of external tools, mainly from R (Ihaka and Gentleman, 1996) and Python (Python Software Foundation, 2015). The software has been integrated into the GROMACS environment (Lindahl et al., 2001; Van Der Spoel et al., 2005). The user must compile it specifically.

GSATools was used to finely analyse the NtrC receiver domain and its homologs CheY and FixJ. For this purpose, different conformations of the protein extracted from a MDs simulation were encoded. The distributions of SA strings were used to compute different mutual information matrices using information theory. Remarkably, they were able to detect allosteric signal transmission from protein dynamics (Pandini et al., 2012). They also applied this methodology to a larger set of related proteins to show how evolutionary conservation and binding promiscuity have opposite effects on intrinsic protein dynamics (Fornili et al., 2013). Other examples are provided in Section 4.

These innovative approaches have been useful to study specific proteins implicated in pathologies and diseases. They are also sufficiently powerful to analyze large datasets of protein structures using automated pipelines. To summarize, SAs provide new visions for the analyses and prediction of protein structure flexibility. Different examples will be detailed in the following sections.

## Duffy antigen/chemokine receptor (DARC) protein

Using the approaches described above, we analyzed conformations of different proteins implicated in pathologies. A very first study was done on predicting flexibility of loops in the Duffy antigen/receptor for chemokine (DARC) protein (Cutbush and Mollison, 1950; Compton and Haber, 1960). DARC is a transmembrane protein localized in the plasma membrane of red blood cells. It is a non-specific receptor for several chemokines (Allen et al., 2007); it is also named atypical chemokine receptor 1, Fy glycoprotein (FY), or CD234 (Cluster of Differentiation 234). The transmembrane chemokine receptors comprise two main families, defined by differences in their ligands. Indeed, chemokines can contain either two consecutive Cysteines (the CC chemokines) or two adjacent Cysteines with one amino acid in-between (the CXC chemokines). Furthermore, the two families of chemokine receptors have a specific linear sequence motif in their C-terminus region that enables signal transduction. In contrast, DARC lacks the specific motif, thus showing a specific difference coming probably from a distinct evolution.

This protein is also known as the receptor for the human malarial parasites *Plasmodium vivax* and *Plasmodium knowlesi* (Miller et al., 1975, 1976). Polymorphisms of DARC are the basis of the Duffy blood group system. While malaria is the most important sickness associated with DARC (Guerra et al., 2006; Cutts et al., 2014), DARC plays also a role in numerous other diseases, such as HIV and cancer, and risk factor associated with many other diseases is emerging (Liu et al., 1999; Horne and Woolley, 2009).

Like most transmembrane proteins, no experimental structure of DARC is currently available (de Brevern et al., 2005). We designed a structural model based on a comparative modeling approach. Using rhodopsin (the only available related structure at this time) as a structural template (a simple alignment showed a very low sequence identity value of 12%, e.g., close to a random value), we carefully built different structural models, based on a hierarchical and iterative procedure. A first step was to predict using more than 10 methods the positions of the 7 transmembrane helices along the sequence. From this initial and rough model, helices of DARC were aligned with rhodopsin helices assigned from the 3D structure. The same methodology was used for the loops, a complete alignment was generated using helices and connecting loops. A specific treatment was done for N- and C-termini region, combining Protein Blocks prediction (de Brevern et al., 2004; Etchebest et al., 2005) with threading approaches.

Experimentally, 40 Alanine mutants had been produced and associations binding constants with CXC-L8 were evaluated (Tournamille et al., 2003, 2005). We used these experiments to assess the quality of our best refined models. From the results, we generated new models by manually changing the positions of helices (and the alignments). Building and refinements were done 10 times until a proper set of characteristics were obtained. In regards to these experiments, *in silico* analysis of protein flexibility has underlined specific characteristics of different epitopes and interaction regions.

**Figure 4:**
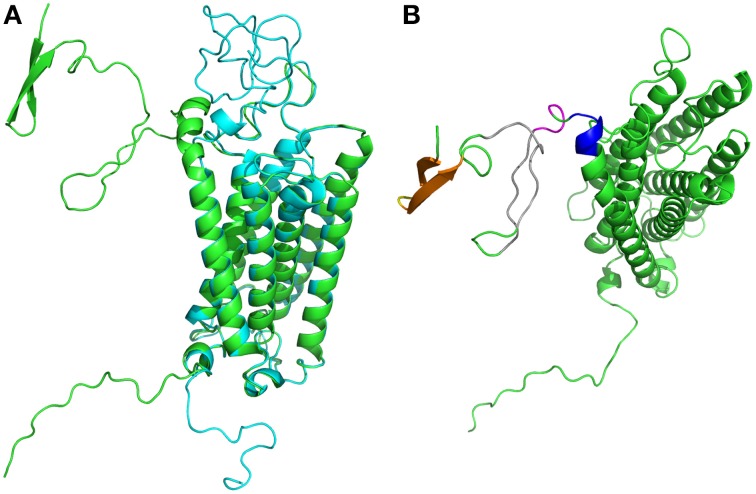
**Duffy Antigen/Receptor for Chemokines structural models. (A)** Are shown the two best structural models obtained (de Brevern et al., 2005), in blue the compact one, in green the located far away. They are near identical on transmembrane domains. **(B)** The different regions with significant local conformational tendencies are shown in other colors. The most important ones are within the first 15 residues (predicted as disordered), with two β-stands (in orange) connected by a short turn (in yellow), later two long regions show some local conformational tendencies to be in extended conformation (in gray color) while the end of this N-terminus is packed in helical conformation (in pink and in violet). Visualization was created by the program PyMOL (http://www.pymol.org, Delano, 2013).

Interestingly, we obtained two different conformations (see Figure 4A) that were both as compatible with experimental data and similarly scored by the few assessment approaches available for transmembrane structural models. Interestingly five years later, an attempt to generate better models with the best available methods was not crowned with success (de Brevern et al., 2009; Smolarek et al., 2010).

It took us one year to build such models (models are available at Model Archive website (http://modelarchive.org/, Schwede et al., 2009). The N_terminus_ is particularly important in the infection by *Plasmodium vivax* (Batchelor et al., 2014). It is nearly 55 residues long and different disorder prediction methods (i.e., DisEMBL, Linding et al., 2003 or PrDOS, Ishida and Kinoshita, 2007), predicted as partially disordered, with the beginning of the sequence as fully disordered.

To evaluate the different conformational states of the Duffy protein, we carried out numerous MDs simulated annealing simulations with the GROMACS software (Lindahl et al., 2001; Van Der Spoel et al., 2005). MD simulated annealing allows a harsh sampling of the conformational space by crossing energetic barriers in an efficient and fast way. Many runs were performed and the different conformations obtained at room temperature were analyzed using Protein Blocks. In practice, we encoded each 3D protein structural model conformation into a 1D string (the length of the protein sequence) using Protein Blocks. Then, we computed, the number of times each PB was observed for each position. Positions with a high frequency of a single PB exhibit no local change, while some others positions exhibit local deformations that require a more in-depth analyses. Few variations could be observed in the helical regions (PB *m* and encompassing PBs) that were weakly restrained with harmonic forces. Instead, loops sampled large regions of the conformational space. A very interesting result was observed for the N_terminus_ region, and especially the distal region. In contrast to what was suggested by disorder predictors, this region was not a random coil region, but in fact a small β-sheet composed of two β-strands (PBs *d* and encompassing PBs, seen in orange on Figure 4B), connected by a short turns (in yellow). In the β-sheets, some positions, e.g., 12 and 13, were invariant. Likewise, the closest region to the first helix was more constrained than expected and not disordered (in pink and violet). Even the central regions (in gray) showed some tendencies to be structured. It was a striking example of a complex series of conformations which cannot be analyzed for instance through classical secondary structure (Kabsch and Sander, 1983).

A second example on DARC loops was the last extra-cellular loops for which a specific and constrained loop conformation was observed. Remarkably, this unexpected conformation explains a “lethal” mutation for the binding of CXCL8. It was the first time a structural alphabet was used to analyze the dynamics of a protein structures or structural model.

## Human integrin α2bβ3

In another project, we were interested in integrins, a large family of cell surface receptors involved in cell—cell or cell—matrix adhesion. Integrins are type I membrane glycoproteins composed of two distinct α and β subunits. Each subunit has a large extracellular region (composed of multiple structural domains), a trans-membrane segment and a short intracellular domain. Integrins interact with cell cytoskeleton and mediate bi-directional trans-membrane signal transduction. These receptors are expressed in vertebrate, but also in lower metazoans including sponges, nematode *Caeorhabditis elegans* and fruitfly *Drosophila Melanogaster*. In mammals, 18 α and 8 β subunits assemble in 24 distinct integrin complexes. Integrins play critical roles in many physiological processes like hemostasis, immune response, leukocyte trafficking, development and angiogenesis or in pathology like cancer. In human, they are responsible for many diseases from genetic or immune origins. They also make effective targets for drug therapies in thrombosis and inflammation. Furthermore, integrins are binding sites for many viruses and bacteria (Hynes, 2002; Takada et al., 2007).

In regard to these various characteristics, integrins have been extensively studied over the past decades. Especially, structural analyses have provided substantial insights to explain functional mechanism(s). In 2004, the first structure of the extracellular domain of αVβ3 integrin, a vitronectin receptor found in platelets, was proposed (Xiao et al., 2004). Then, several structures of αVβ3 but also of αIIbβ3 integrin (Zhu et al., 2008), a fibrinogen receptor involved in platelet aggregation, were resolved in different activation states. Molecular models for both trans-membrane and cytoplasmic domains were also proposed. Thus, it opens the way to investigate impact of mutant using *in silico* mutagenesis.

Hence, we examined the effect of the β3-Leu253Met substitution of αIIbβ3 complex in patients with Glanzmann thrombasthenia (Jallu et al., 2010), a rare bleeding disorder characterized by an impaired platelet aggregation (George et al., 1990). For the first time, we showed that residue Leu253—localized at the interface of the complex—is playing a major role in the stability of αIIbβ3. Nonetheless, structural models reflecting static specific states do not depict structural dynamics accompanying the various aspects of integrin functions. For instance, when integrins are activated by substrates, large conformational changes are observed. Analyses of static structures (e.g., B-factor, electrostatics), give only a limited view of the protein complex behavior, contrary to MDs simulations which are able to some extent, to reproduce the inner dynamics of protein structures.

α and β subunits of integrins are associated to rigid, flexible and even disorder properties (such as Duffy protein presented in the section above). We ran independent MDs simulations on different systems, i.e., the wild type but also variants and mutants, using GROMACS MDs package (Van Der Spoel et al., 2005) to examine specific regions of αIIbβ3. We observed different opposite behaviors depending on the region and mutants studied.

Hence, we studied the Cab3^a+^ alloantigen resulting from a Leu841Met substitution in the αIIb chain. This polymorphism might result in severe life-threatening thrombocytopenias. Cab3^a+^ corresponds to a Leu841Met mutation. We evaluated the flexibility by using *N*_eq_ index and found that this polymorphism locates in a very flexible sequence in the wild type (with a *N*_eq_ > 4), but the mutation did not modify the *N*_eq_ behaviors (Jallu et al., 2013). Moreover, no change in the secondary structure content, neither the PBs adopted by residues of encompassing sequences change. Hence, intriguingly, this substitution would have little effect, if any, on the backbone structure of the peptide 829–853. It must be noticed that disorder prediction does not show this region has flexible property, i.e., prediction with IUPred (Dosztanyi et al., 2005) or DisEMBL (Linding et al., 2003).

In Caucasian population, the Human Platelet Alloantigenic (HPA) system 1 is involved in most neonatal thrombocytopenias (NAITP) and post-transfusion purpura (PTP) (Espinoza et al., 2013). The HPA-1 system results from a Leucine to Proline substitution in position 33 of the β3 chain (alleles HPA-1a and HPA-1b, respectively) in platelet αIIbβ3 integrin (Jallu et al., 2012). Alloantibodies to the HPA-1a variant can induce very severe immune thrombocytopenia (Espinoza et al., 2013). Furthermore, the Pro33 allelic variant of β3 is considered as a risk factor of thrombosis in patients with cardiovascular diseases.

To compare the HPA-1a and -1b variants, we have proposed for the first time to use a combination of standard analysis of flexibility (namely Root Mean Square Fluctuation, RMSF) and Protein Blocks analyses. MD simulations have revealed that (i) the Leu33Pro substitution of the β3 knee (a domain of β3 integrin chain) leads to adverse structural effects not highlighted by static models; and (ii) that these alterations can explain the increased adhesion potential of HPA-1b platelets to fibrinogen and the possible thrombotic risk associated with the HPA-1b phenotype (Jallu et al., 2012). These molecular simulations also support a novel structural explanation for the epitope complexity of the HPA-1 antigen (Jallu et al., 2012).

Although not yet known to be involved in an alloimmune response, a third variant discovered more recently and characterized by a Valine in position 33 of β3, was also examined. Analyses of the protein flexibility properties can mainly explain the variable reactivity of anti-HPA-1a alloantibodies. This result suggests that dynamics plays a key role in the binding of these alloantibodies. Unlike the L33P substitution which increases the local structure flexibility, the L33V transition would not affect the local structure flexibility, and consequently the functions of αIIbβ3 (Jallu et al., 2014). Although, this region is considered as rigid by disorder prediction, both RMSF and PBs analysis shows a high mobility. This behavior may be explained by a local rigidity, surrounded by deformable regions.

**Figure 5:**
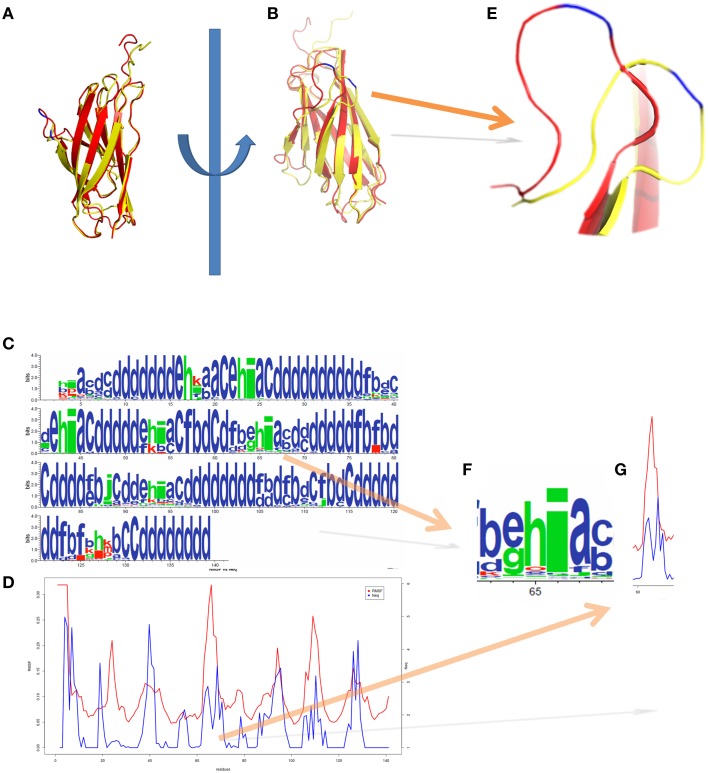
**Integrin Calf-1 domain simulation using PBxplore**. **(A,B)** show the superimposition of two distinct snapshots (in red and in yellow) extracted from a molecular dynamics. **(C)** Shows the PB distribution in terms of WebLogo (Crooks et al., 2004) obtained thanks to PBxplore, and **(D)** represents the superimposition of *N*_eq_ and RMSF for the whole domain. **(E)** Zoom on the loop containing the residue 66 of Cab-1 domain (blue) which shows a dedicated **(F)** PB pattern *i* with a **(G)** low *N*_eq_ and high RMSF, i.e., a mobile position in a “flexible” region. Visualization was created by the program PyMOL (http://www.pymol.org, Delano, 2013).

Figure 5 represents another MDs simulation focusing here only on the Calf-1 domain (a domain of α2b integrin chain), using same parameters as before. Simulations were analyzed through PB approaches underlining its interest for flexibility studies using PBxplore. Figures 5A,B show the superimposition of two distinct snapshots (in red and in yellow) extracted from the MDs simulation. Figure 5C shows the frequency of PBs at each position, calculated along the MD trajectory, and represented as a WebLogo graphic (Crooks et al., 2004) obtained with PBxplore. WebLogo (Crooks et al., 2004) summarizes this information with an entropy of every PBs at each position. Figure 5D is the superimposition of *N*_eq_ and RMSF. Interestingly, even though some regions show similar tendencies, namely large RMSF associated with large *N*_eq_, other regions exhibit different and even opposite tendencies. For example, focusing on the residues near position 66 of Calf-1, the RMSF given Figure 5G, is the highest one (in blue on Figure 5E) as highly flexible, but it is not the case as the *N*_eq_-values for this residue is not high. Therefore, these residues appear to be a mobile region between two deformable regions. This example confirms the interest to examine *N*_eq_ index beside RMSF because each measure brings related but different information on flexibility.

**Figure 6:**
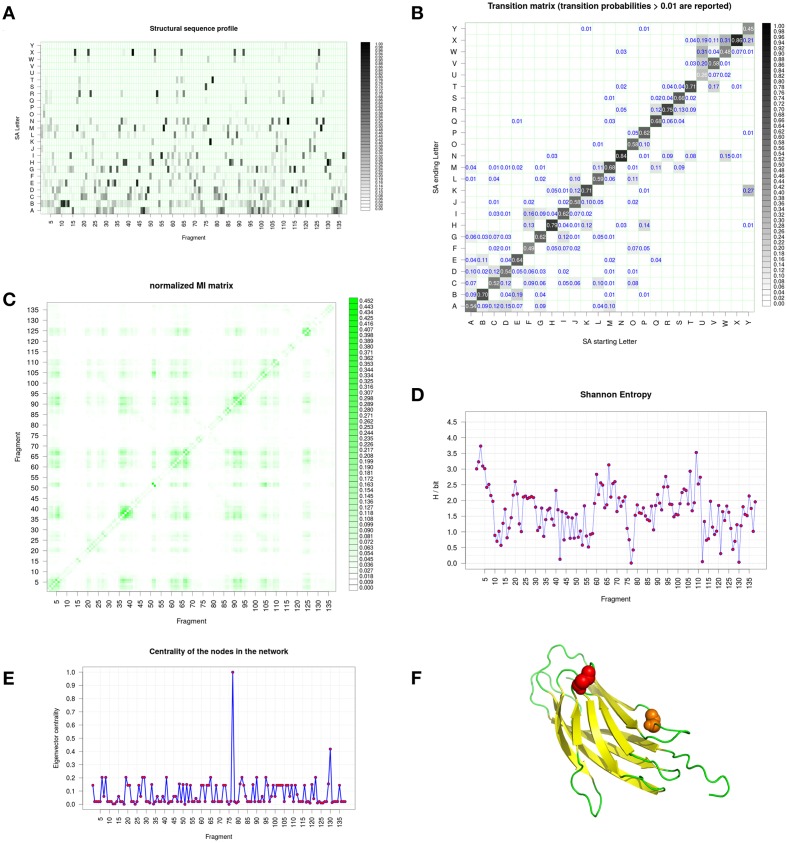
**Integrin Calf-1 domain simulation analyses using GSATools. (A)** shows the structural alphabet distribution during the simulation, i.e., the sequence profile of the alignment, **(B)** the transition probability matrix for the transitions between SA letters, which reflect local conformational changes, **(C)** the Mutual Information (MI) matrix describing the correlation of local conformational changes among the protein's fragments, **(D)** the Shannon entropy per position, and **(E)** the graph representation of the correlated local motions from the MI matrix, with the eigenvector centrality, which describes the relative importance of the nodes in the network. **(F)** Is a visualization created by the program PyMOL (http://www.pymol.org, Delano, 2013) of the two most important peaks underlined by **(E)**.

Figure 6 shows the structural alphabet distribution during the simulation obtained with GSATools (see Section The Different Views of Protein Structures). The most frequent letters seen (in black) are from the beginning of the alphabet, underlining its all-β composition (Figure 6A). The decomposition by this SA shows a large number of conformational changes at each position of the sequence. Only few positions, e.g., 10, 131, and 44 represented by B, H, and X letters, respectively, remained unchanged during the Calf-1 simulations. The transition probability matrix calculated between SA letters (Figure 6B) reflects how the local structure changes occur. Along the diagonal, high values are found, the highest ones being for letters N and X while the lowest ones being for letters U and Y The Mutual Information (MI) matrix presented in Figure 6C describes the correlation of local conformational changes among the protein fragments. Significant off-diagonal values are found but actually they correspond to strands forming β-sheets. Hence, in contrast to the examples detailed in (Pandini et al., 2012, 2013), the all-β conformation of the protein impedes to enlighten long-range correlations, except between β –strands close in 3D and found all along the protein sequences. The Shannon entropy per position shows quite similar profile between β –strands (mainly between 1.0 and 2.5 bits). All the lowest values correspond to residues inside loops. One of the most interesting features of GSATools is the graph representation of the correlated local motions from the MI matrix; it describes the relative importance of the nodes in the network useful to analyze allosteric behaviors. Figure 6F is a visualization of the two most important peaks underlined, they are found far away from the rigid β-sheet region.

## Protein complexes and allostery

It is well documented that protein–protein interactions are often guided by flexibility (Jones and Thornton, 1996; Salwinski et al., 2004) and that alternative conformations can have a significant influence on the binding process. It is why predicting the structure of a complex using the unbound structures of the partners remains highly challenging, despite a scrutinizing examination of the amino acid composition of the interface (Janin et al., 2008). Thus, in most cases, protein structures change during the formation of the complex. The changes can be limited to few side chains motions but can also correspond to major reorganization in the fold. Therefore, we undertook the analysis of the protein–protein complexes in the light of structural alphabet. We compared proteins 3D structures in free form, and as part of larger macromolecular complexes.

**Figure 7:**
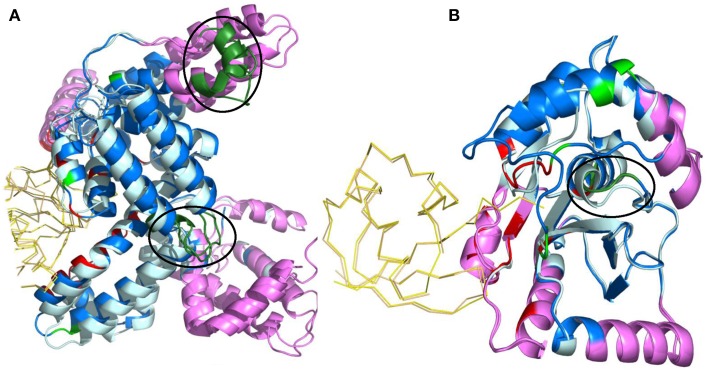
**Normal mode analysis of structural changes in regions of low B-factor far from interface**. The protein containing the region of interest is depicted as cartoon and the interface of the other protein in ribbon. Unbound and bound forms of the protein of interest are in pale cyan and marine blue, respectively. The partner protein's unbound and bound forms are in light orange and yellow, respectively. Interacting residues are in red and non-interacting residues with PB change in green. All regions of interest are marked with a black circle, irrespective of whether they are intrinsically mobile or rigid. Regions identified to be intrinsically mobile according to NMA are in violet. Regions of interest occurring within the intrinsically mobile segments are in dark green. The complexes shown are **(A)** α-actin and Vitamin D - binding protein (PDB code 1KXP, Otterbein et al., 2002) **(B)** Ubiquitin Carboxyl-terminal esterase L3 protein and Ubiquitin complex (PDB code 1XD3, Misaghi et al., 2005). These figures show that non-interacting regions observed to undergo conformational changes upon complexation are usually intrinsically mobile, which is a characteristic of a functional site. Visualization was created by the program PyMOL (http://www.pymol.org, Delano, 2013).

The building of the protein dataset was quite strict leaving only 76 high quality complexes representing very different configurations with free and bound forms (Swapna et al., 2012). Accordingly, structural changes occurring between the free and bound forms of the protein were analyzed using three different measures: the Cα root mean square deviation, the percentage of PB change and a specific PB substitution score. This last score relies on a PB structural substitution matrix that quantifies the cost to replace a given PB by another PB. The more similar the PBs, the more favorable the substitution score. Consequently, this score permits to quantify the conformational change by distinguishing similar PBs from to the most distinct ones. Comparison between unbound and bound forms shows that significant structural rearrangement occurs at the interface but also in regions away from the interface upon the formation of a highly specific, stable and functional complex. For 50% of them, which correspond to signaling proteins, the major changes correspond to allosteric ones, localized far away from the interface. These sites could be associated to mutations known to be involved in multiple diseases such as cancer. PB allows distinguishing here also between large movements, from mobility to deformability or flexibility. Normal Mode Analysis was also performed to gain deeper insights (Swapna et al., 2012). The results obtained for signaling complexes underline the importance of allostery-like structural changes much more than appreciated before (see Figure 7).

Flexibility becomes a critical issue in complexes especially the ones involving intrinsically disordered protein. Fine analyses have shown that disordered proteins can also adopt well-defined conformations in their bound form; their inherently dynamic nature is cast into their complexes (Meszaros et al., 2011). Protein families with more diverse interactions exhibit less average disorder over all members of the family (Fong and Panchenko, 2010). Inter-domain linkers are evolutionarily well conserved and are constrained by the domain-domain interface interactions (Bhaskara et al., 2013). An interesting resource is the ComSin database which provides a collection of structures of proteins solved in unbound and bound form, targeted toward disorder–order transitions (Lobanov et al., 2010).

## Protein/DNA interfaces

Beside protein-protein interactions, which govern many biological functions, fundamental biological processes like transcription also require complex formation, i.e., between protein and DNA. As for protein-protein interaction, complexation can change structures of both partners, but most studies focused on the protein side. Most of protein/DNA interfaces only extend the classical approaches to analyze protein/protein interfaces or protein/ligands interface. For instance, in PDIdb (Ferrada and Melo, 2009) or Biswas and coworkers studies, the interface is classified into core and rim regions, the first one being more sequentially conserved. Biswas and coworkers proposed a new classification scheme for the interfaces based on the composition of secondary structures (Biswas et al., 2009). Beyond this description in terms of regular local structures, Sunami and Kono (2013) conducted a quantitative analysis to understand the conformational changes in proteins when they bind to DNA. They compared DNA-free and DNA-bound forms of proteins and used structural alphabets to describe conformational changes in 4-residue fragments. They found that (i) three specific alphabets appeared in the DNA interfaces, (ii) conformational changes in DNA interfaces are more frequent than in non-interfaces and importantly, (iii) regions involved in DNA interfaces have more conformational variations in the DNA-free form. This study underlines also the importance of intrinsic flexibility of interacting regions to fit into DNA structure.

**Figure 8:**
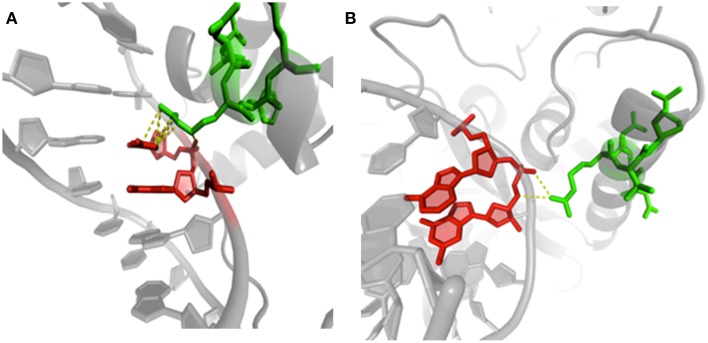
**Examples of protein/DNA interactions. (A)** Structure of human centromere protein B (CENP-B) binding to DNA CENP-B box (PDB code 1HLV, Tanaka et al., 2001). The image highlights contacts between arginine 125 (chain A, green) in PB *m* (regular helix) and cytosine 15 (chain B, red) in ntC 41. **(B)** Details of methionine repressor protein (MetJ) binding to DNA metbox (PDB code 1MJQ, Garvie and Phillips, 2000). The same PB *m* and amino acid residue (arginine 40 in chain H, green) is in contact with guanine 2 (chain K, red) in NtC 13. Visualization was created by the program PyMOL (http://www.pymol.org, Delano, 2013).

Another recent analysis has explored an extensive set of protein/DNA complexes and looked at conformational changes occurring in proteins but also in DNA. Importantly, for both molecules, structural alphabets were used. The alphabet used for describing protein backbone is the Protein Blocks. For DNA, a structural alphabet was obtained using a new approach of registering torsion angles of a dinucleotide unit combined with Fourier averaging and clustering (http://www.dnatco.org/, Svozil et al., 2008; Cech et al., 2013). These structural alphabets describe biopolymer conformations at greater detail than the 3-state protein secondary structure and basic DNA structural types such as A, BI and BII. Figure 8 shows an example of different conformations. This study compared structural features of the protein/DNA interface with the features of non-interacting parts of protein and DNA molecules. Clear differences in preferences for occurrences of local protein and DNA conformations were observed. Specific preferences were underlined between complexes containing various types of proteins such as transcription factors and nucleases. Minor DNA conformers are often significantly enriched at the interface so that the ability of DNA to adopt non-canonical conformers, rare in naked DNA, is clearly essential for the recognition by proteins. Rare DNA conformations introduce significant deformations to the DNA regular structure. The occurrence of these rare forms was estimated and characterized enabling a better understanding of the role of non-B-DNA structures. A critical feature was the distinct interaction patterns for the DNA minor groove relative to the major groove and phosphate, and the importance of water-mediated contacts. Indeed, water molecules mediate a proportionally largest number of contacts in the minor groove and form the largest proportion of contacts in complexes of transcription factors (Schneider et al., 2014). It corroborates to previous researches on the importance of mobility of such water molecules (Luo et al., 2011; Russo et al., 2011).

**Figure 9:**
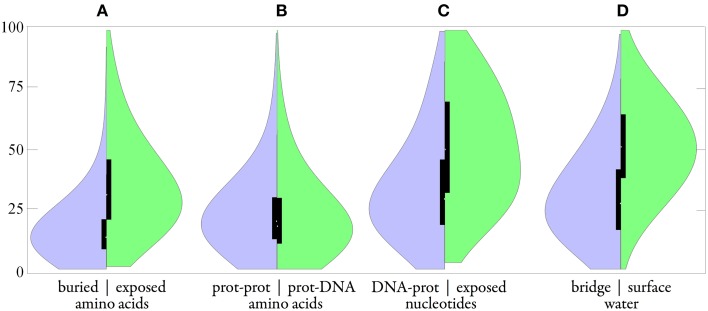
**Distributions of B-factors in the group of protein-DNA complexes (165 structures with crystallographic resolution 1.9 Å and better)**. Smooth plot **(A)** compared buried amino acid (left in purple) vs. exposed aa (right in green); **(B)** protein-protein aa vs. protein-DNA aa; **(C)** DNA-protein nucleotide vs. exposed nt; **(D)** bridge water vs. surface w. Black boxes show the second and third quartiles; the white spot indicates the median.

The above-discussed analyses pointed to some remarkable features about the protein/DNA interfaces, so that we performed a more specific analysis of the protein and DNA dynamics based on crystal structures. The analysis of B-factors (Schneider et al., 2014) showed that the dynamics of biopolymer residues, amino acids and nucleotides, as well as ordered water molecules is first of all a function of their neighborhood: amino acids in the interior of proteins have the tightest distribution of their displacements, residues forming the biopolymer interfaces (protein/protein or protein/DNA) intermediate, and residues exposed to the solvent the widest distribution (Figure 9). This general picture is best pronounced for structures with the highest crystallographic resolution since discrimination of different types of residues in structures becomes unclear with lower crystallographic resolution. Besides, amino acid residues in the protein core display a unique feature: their backbone and side chain atoms have virtually identical B-factor distributions. The protein core is therefore extremely well packed leaving minimum free space for atomic movements. B-factors of water molecules bridging protein and DNA molecules were surprisingly significantly lower than B-factors of DNA phosphates; in opposite, solvent-accessible phosphates were extremely flexible. An unexpected conclusion of this analysis is that a part of the observed trends could be due to improper refinement protocols that may need slight modifications (Schneider et al., 2014). Hence, the B-factors of high-resolution structures reflect the expected dynamics of residues in protein–DNA complexes but the B factors of lower resolution structures should be treated cautiously. Based on such kinds of ideas, Vriend proposed a dedicated dataset of refined B-factors (http://www.cmbi.umcn.nl/bdb/, Touw and Vriend, 2014).

## PTMs

**Figure 10:**
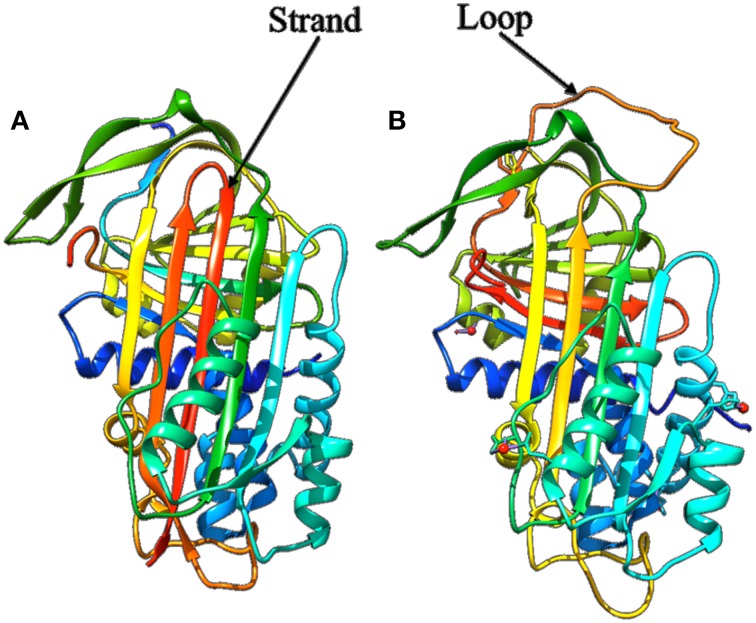
**Structure of the alpha 1 antitrypsin. (A)** The cleaved form after complexation with protease (PDB code 1EZX, Huntington et al., 2000) showing the strand inserted in the β-sheet after cleavage, **(B)** the uncleaved form (PDB code 1HP7, Kim et al., 2001) showing the wild whole loop.

As seen in the previous sections, protein flexibility is essential for interactions between proteins and ligand, nucleic acid, or protein partners. Apart from interaction with partners, chemical modifications like formation or breaking of covalent bonds, can impact structural and dynamics properties. One of the most spectacular examples is depicted by the serpin family members when they interact with the protease (see Figure 10, Huntington et al., 2000; Kim et al., 2001). An initial large conformational change, consecutive to the cleavage of the reactive center of the serpin by the protease, occurs. The loop involved in the cleavage moves, folds as a β-strand that inserts between the other strands of the β-sheet composing the serpin protein core. The two proteins are tightly linked, which significantly affects the protease that looses more than 30% of its structure.

Among chemical modifications, post-translational modifications (PTMs), like phosphorylation, play a major role in many biology processes. Integrins, for example, can be activated consecutive to phosphorylation. The impact of these modifications on the structure and the dynamics of proteins is thus of particular interest.

Recent studies have shown that PTMs have significant effects on the protein conformations and on their flexibility. Hence Xin and Radivojac used 3D structures from the PDB and studied the conformational heterogeneity of protein structures corresponding to identical sequences in their unmodified and modified forms (Xin and Radivojac, 2012). They demonstrated that PTMs induce conformational changes at both local and global level, but with a limited impact. Accordingly PTMs would affect regulatory and signaling pathways (Nussinov et al., 2012; Xin and Radivojac, 2012) by subtle but common mechanisms of allostery. Some prediction approaches and are included into dedicated databases (Matlock et al., 2015), but few analyzed precisely the whole PTMome.

This led us to conduct a deep analysis of structures of the same protein with or without PTMs. As an example, we selected 157 PDB chains of the human Cyclin-dependent kinase 2 (*UniProt AC: P24941*) in complex form, and 222 PDB chains of unbound monomer. Based on data from PTM-SD (Craveur et al., 2014), a database of structurally solved and annotated post-translational modifications, 112 chains among the 157 complexes, present a phosphorylated threonine at position 160 in the structure of the kinase. As described in Table 1, we compared the backbone flexibility of three different cases: unbound kinase, kinase complex, and phospho-Thr160 kinase in complex.

**Table 1:** **Composition of structural complexes involving the Cyclin-dependent kinase 2**.

	**UniProt** **AC** **in** **complex**	**Nbr** **of** **complex**	**Protein** **name** **and** **organism**
	**COMPLEX WITH PHOSPHORYLATION ON Thr 160 IN P24941**		
P24941	P14635	1	G2/mitotic-specific cyclin-B1 Homo sapiens (Humain)
Cyclin-dependent kinase 2	P20248	74	Cyclin-A2 Homo sapiens (Humain)
Homo sapiens (Human)	P24864	1	G1/S-specific cyclin-E1 Homo sapiens (Humain)
	P30274	22	Cyclin-A2 Bos taurus (Bovin)
	P51943	8	Cyclin-A2 Mus musculus (Souris)
	Q16667	1	Cyclin-dependent kinase inhibitor 3 Homo sapiens (Humain)
	P20248 + Q99741	4	Cyclin-A2 Homo sapiens (Humain) + Cell division control protein 6 homolog Homo sapiens (Humain)
	P20248 + P46527	1	Cyclin-A2 Homo sapiens (Humain) + Cyclin-dependent kinase inhibitor 1B Homo sapiens (Humain)
	Total	112	
	**COMPLEX WITHOUT PHOSPHORYLATION ON Thr 160 IN P24941**		
	P20248	42	Cyclin-A2 Homo sapiens (Humain)
	P61024	1	Cyclin-dependent kinases regulatory subunit 1 Homo sapiens (Humain)
	P89883	2	V-cyclin of Murid herpesvirus 4
	Total	45	

**Figure 11:**
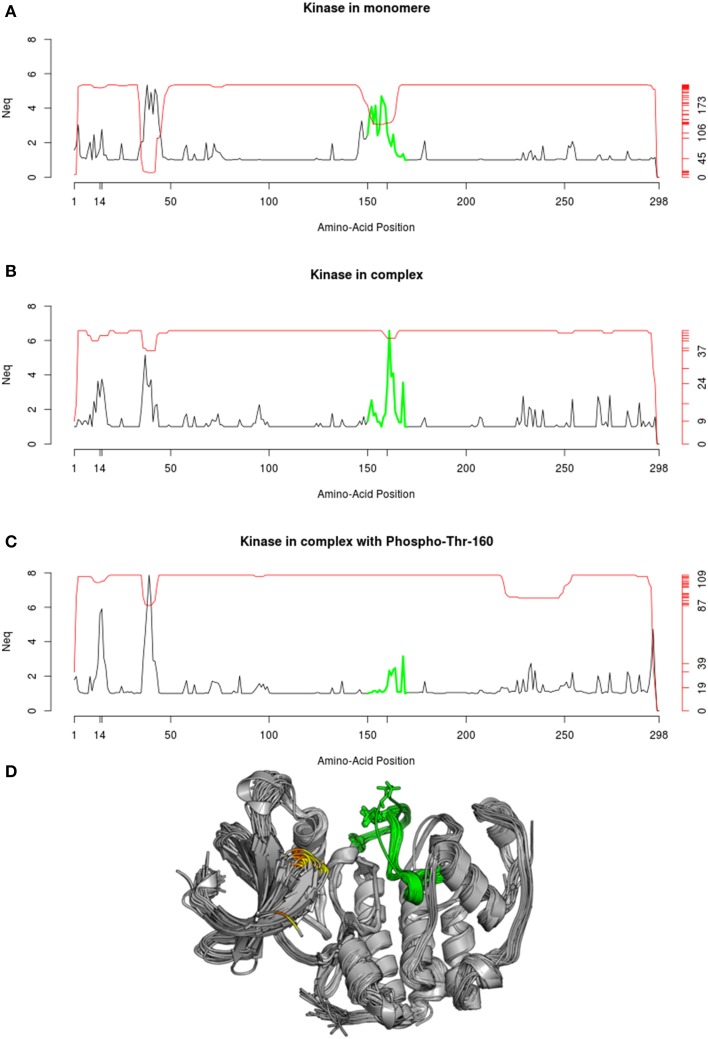
***N*****_eq_ profile of Cyclin-dependent kinase 2**. The *N*_eq_ profile is given in each case: **(A)** for structure of kinase found in monomer, **(B)** found as part of a complex, **(C)** found in complex with a phosphorylation solved in the Thr-160. **(D)** The superposition of the 112 PDB chains used to compute the *N*_eq_ profile in **(C)** is shown. The corresponding green region is highlighted, and positions 14 and 15 are, respectively, indicated in yellow and orange. Protein visualization was created by the program PyMOL (http://www.pymol.org, Delano, 2013).

Comparison of the three *N*_eq_ profiles, shown in Figure 11, highlights significant differences in local flexibility of the kinase structures. Figure 11A shows that, when kinase is in unbound form, the polypeptide chain presents a flexible fragment (colored in green), which corresponds to a large loop. When complex is formed (Figure 11B), this loop is placed at the interface and leads to stiffening of its edges and higher flexibility in the neighborhood of Thr-160. This change is characterized by a diminution and an increase of *N*_eq_-values, respectively. Finally, when the Thr-160 is phosphorylated (Figure 11C), the green region becomes comparatively rigid, which results to limited flexibility (*N*_eq_ ≤ 3.16). However, another region in kinase (position 8 to 18) is associated with increasing flexibility. When the complex is forming, the *N*_eq_ range in this area increases from (1;2.77) to (1;3.76), and secondly, when the phosphorylation is in place, the range increases to (1;5.91). Interestingly, this region corresponds to the neighboring positions of two other phosphorylation sites, at Thr-14 and Tyr-15. It is important to note that these phosphorylations were absent in the structures used here for the *N*_eq_ computation.

In a functional point of view, the phosphorylation in position 160 is known to promote the activation of the kinase, while the phosphorylation of position 14 and 15 slightly reduce its activity (Gu et al., 1992). Thereby, the changes in flexibility observed at these 3 phosphorylation sites, could reflect that the activity of the kinase is regulated by a mechanism of complementary rigidity/flexibility of local protein backbone, which could be related to allosteric effects.

The red line plotted in Figure 11 represents the number of available structural data for each position. Interestingly, the green region in Figure 11 is proportionally less resolved when kinase is in monomer than when it is in complex, and even more solved when the Thr-160 is phosphorylated. This observation emphasizes that the decrease of flexibility in this region facilitates the resolution of the structures. Several structures of the same protein present specific regions that are disordered in some crystals and ordered in others. These regions were defined by Zhang and collaborators as “Dual Personality Fragments” (Zhang et al., 2007), and the corresponding fragment of the green region in Cyclin dependent kinase was the emblematic example used by Zhang et al. (2007) to defined DPF. In the same way, the region between positions 35 to 45 were also identify as DP fragments.

## Prediction of protein flexibility

The growing gap between the number of protein sequences and the number of atomic structures imposes to resort to alternative approaches to gain structural and dynamics information. They are mainly based on crystallographic B-factor analyses. It is often seen that crystallographic B-factors are a mix of properties, dynamics being one of them. Recent approaches show that NMR spectroscopy provides an ever increasing amount of dynamics data, going well beyond the simple thermal vibrations (Powers et al., 1993; Palmer, 2001; Olsson et al., 2014). None of them can describe all the important flexible movement or even disorder. Hence, it must be taken into account that everything in protein dynamics cannot be assessed based on a single view Prediction methods are therefore of particular importance. Flexibility prediction from sequences started as a Boolean prediction, i.e., rigid or flexible, using simple statistical analyses of B-factor values (Karplus and Schulz, 1985; Vihinen et al., 1994). Following developments combined evolutionary information to different machine learning methods, such as Artificial Neural Networks (Schlessinger et al., 2006), support vector regression coupled with random forest (Pan and Shen, 2009), and support vector machines (Kuznetsov, 2008; Kuznetsov and McDuffie, 2008). Additional sources of information were progressively take into account, rather than X-ray B-factors, as Nuclear magnetic resonance data (NMR) (Trott et al., 2008; Zhang et al., 2010), dihedral angles and accessibility (Hwang et al., 2011), or computational data from Normal Mode Analysis (Hirose et al., 2010). At last, some methodology, dedicated to predict protein disorder were also developed and designed to high flexibility prediction (Galzitskaya et al., 2006; Mamonova et al., 2010; Jones and Cozzetto, 2015). Recent approaches are quite complex like (i) the DynaMine webserver (http://dynamine.ibsquare.be/, Cilia et al., 2013, 2014), DynaMine predicts backbone flexibility at the residue-level in the form of backbone N-H S^2^ order parameter values learnt from NMR data, or (ii) as a predictor which used relative solvent accessibility (RSA) and custom-derived amino acid (AA) alphabets. The prediction is done in two-stage linear regression model that uses RSA-based space in a local sequence window in the first stage and a reduced AA pair-based space in the second stage as the inputs (Zhang and Kurgan, 2014).

We also proposed prediction of protein flexibility of an amino acid sequence using the potentialities of SA prediction. The approach is not only innovative through the use of local protein conformations, but also with specific definition of flexibility. Flexibility is often defined based on α-carbon B-factor values obtained from X-ray experiments. As mentioned above, these data reflect protein flexibility, but may also be prone to experimental and systematic biases. Hence, flexibility was considered with X-ray B-factor descriptors and the RMSF observed in MDs simulations, which is calculated from the amplitude of atom motions during simulation. Both descriptors were combined to define and to examine flexibility classes of SA.

This dedicated prediction method is divided in two steps: first an SA prediction from sequence, and second a flexibility prediction from the SA predicted. The SA used in this method is the LSP (see Section The Different Views of Protein Structures). They consist of 120 overlapping structural classes of 11-residue long fragments (Benros et al., 2006), which encompass all known local protein structures and ensure good quality 3D local approximation. The major advantage of this library is its capacity to capture the continuity between the identified recurrent local structures (Benros et al., 2009). We can notice that is quite difficult to have a good correlation between theoretical results to actual experiments. With LSPs, we have shown that they have on average a correlation > 0.9 with B-factors.

Relevant sequence–structure relationships were also observed and further used for prediction. Briefly, LSP prediction is based on SVM training. With the LSP prediction, a Confidence Index (CI) that is based on the discriminative power of the SVMs is provided. The higher CI, the better the prediction rate is. The prediction rate reaches 63.1%, a rather high value given the high number of structural classes (Bornot et al., 2009).

In a second step, we considered the two descriptors for quantifying protein dynamics, X-ray B-factors and RMSF. They were combined to define 3 flexibility classes of LSPs: rigid, intermediate and flexible. Then for each 11-residue long target sequences, the SA prediction provided a list of five possible LSP candidates. Based on the previously defined flexibility classes of these structural candidates, the prediction of target flexibility is made. Interestingly, the prediction rate is slightly better than the one of PROFbval (Schlessinger et al., 2006) that was optimized for only two classes.

Hence, the originality of the method lies (i) in the use of a combination of B-factors and RMSF for quantifying protein dynamics, (ii) in prediction of flexibility through SA prediction of LSPs, and (iii) in prediction of three classes of flexibility, which are usually limited to two. The method is implemented in a web server named PredyFlexy (http://www.dsimb.inserm.fr/dsimb_tools/predyflexy, de Brevern et al., 2012), in which the users have access to a confidence index (CI) for assessing the quality of the prediction rate.

## Conclusion

The protein structure organization is characterized by a conformational arrangement of repetitive structures (secondary structures, i.e., α-helices, β-sheets and coils/loops). Static observation of protein organization has revealed some of their essential properties, i.e., active sites are generally found at the protein core in which residues are well packed and mainly hydrophobic, while the surface residues, exposed to solvent or to another partner(s) (protein, DNA), are more flexible because less constrained than the core. The function of proteins and their interaction mechanism need some flexible properties that are considerably more complex than this simplistic binary view. By exploiting various structural data sources and by developing different computational methods (B-factor, NMR data, MDs Simulation, NMA, …) dynamics of proteins turn out to cover a large spectrum of conformational changes (combined by mobility of rigid fragment and deformability of backbone), by the existence of intrinsic disorder region, by allosteric effect… Some of these flexible mechanisms need structural reorganization at a local level. Thus investigation of protein flexibility requires a more local and complex description of protein structures than the classic representation.

In this review we have illustrated using numerous examples (DARC protein, Human integrins, Protein Complexes, Protein/DNA interfaces, Proteins with Post-Translational Modifications) how the approaches, based on Structural Alphabets, are a valuable tool to study flexibility at this level.

From our experiences with these examples, we can state that the use of SAs allows to tackle and address the important problem of the comparison of an ensemble of protein conformations. Indeed, in a recent paper, Scott and Strauss (Scott and Straus, 2015) underlines the bias related to the use of RMSD, which needs beforehand an optimally superimposed approach often remains as rigid bodies. They proposed an elegant method, fleximatch, of protein structure comparison that tries to take flexibility into account. As it was done for protein superimposition methods (Yang and Tung, 2006; Tung et al., 2007; Tung and Yang, 2007; Le et al., 2009; Budowski-Tal et al., 2010; Gelly et al., 2011; Leonard et al., 2014), SA is an efficient approach, not considering proteins as rigid bodies. We underline the interest of our approach based on Protein Blocks with the PBxplore tools (https://github.com/pierrepo/PBxplore, in preparation) or GSAtools (http://mathbio.nimr.mrc.ac.uk/wiki/GSATools, Pandini et al., 2013) in other cases. The use of SAs and the development of associated metrics such as *N*_eq_ is required to study the details and begin to understand the complexity of protein flexibility. It allows discriminating flexibility from mobility and deformability, which is not currently considered by other available methods. Nonetheless, it also had drawbacks as no simple threshold will guide the researcher to point out that certain segment is THE highly flexible part and not the other, same as for RMSF. In the same way, use of information theory with GSATools also requires expertise. Moreover, as SA represents a simplification of the 3D description, its results can be compared to the Normal Mode Analysis based on Elastic Network Model (Suhre and Sanejouand, 2004; Tiwari et al., 2014; Eyal et al., 2015) that are efficient to define large movement. However, changes at a finer level such as side chain rotameric states or minor changes in the backbone (but essential for the biological functions) are more difficult to handle. Here as always, a good knowledge of the biological system is essential as a correct definition of the scientific question and its scale (Buehler and Yung, 2009).

To conclude, we can find that all these approaches are suitable for highlighting both flexible and rigid parts of a protein from structures derived from NMR, X-ray diffraction or molecular simulation.

## Author contributions

Section Introduction: PC, APJ, JE, TJN, FN, JR, CE, NS, JCG. Section The Different Views of Protein Structures: PC, APJ, JE, TJN, MG, SL, PP, GF, JR, AG, LSS, RMB, JB, ST, JC, BS, CE, NS, JCG. Section Duffy Antigen/Chemokine Receptor Protein: NS, OB, CE. Section Human Integrin α2bβ3: APJ, NS, MG, PP, JR, JB, ST, VJ. Section Protein Complexes and Allostery: PC, APJ, JE, LSS, RMB, NS. Section Protein/DNA Interfaces: JC, BS, JCG. Section PTMs: PC, JE, TJN, FN, SL, GF, JR. Section Prediction of Protein Flexibility: PC, APJ, JE, SL, GF, AG, CE, JCG. Section Conclusion: PC, APJ, GF, CE, NS. AdB conceived the review and participated in all the different sections.

### Conflict of interest statement

The authors declare that the research was conducted in the absence of any commercial or financial relationships that could be construed as a potential conflict of interest.
